# The fundamental frequencies of our own voice

**DOI:** 10.1098/rsos.241081

**Published:** 2025-02-19

**Authors:** Hakam Neamaalkassis, Yves Boubenec, Christian Fiebach, R. Muralikrishnan, Alessandro Tavano

**Affiliations:** ^1^ Department of Cognitive Psychology, Max Planck Institute for Empirical Aesthetics, Grüneburgweg 14, Frankfurt a. M. 63122, Germany; ^2^ Département d’Études Cognitives, École Normale Supérieure, PSL Research University, CNRS, 29 rue d’Ulm, Paris 75005, France; ^3^ Department of Psychology, Goethe University Frankfurt, Theodor-W.-Adorno-Platz 1, Frankfurt a. M. 60323, Germany

**Keywords:** change deafness, auditory attention, attentive filters, corollary discharge, subjective perception, self-generation

## Abstract

Own actions send a corollary discharge (CD) signal, that is a copy of the planned motor programme, to sensory-specific brain areas to suppress the anticipated sensory response, providing a neural basis for the sense of self. When we speak, the sensory consequences of the fundamental frequency ($f_{\text{0}}$) of our own voice, generated by vocal fold vibrations, are suppressed. However, due to bone/air conduction filtering effects, the $f_{\text{0}}$ we self-generate is measurably different from the $f_{\text{0}}$ we subjectively perceive as defining our own voice. Using an auditory change deafness paradigm, we parametrically tested the sensitivity to auditory change in the frequency neighbourhoods of objective and subjective own voice pitches and found that participants experience change deafness for both to a similar extent, relative to a control pitch condition. We conclude that when we listen attentively, we are likely to filter out small pitch changes in the vicinity of our own objective and subjective voice $f_{\text{0}}$, possibly as a long-term consequence of speaking-induced suppression mechanisms integrated with individual, perceptual bodily priors.

## 1. Introduction

Complex organisms need to distinguish self-generated from external sources of information. One way for the brain to solve this problem is to send a corollary discharge (CD) signal, that is a copy of the motor programme for a planned action, to sensory-specific brain regions, in order to attenuate the response to self-generated sensory input [1]. When we speak, we attenuate the cortical auditory responses to self-generated speech features, such as the fundamental frequency ($f_{\text{0}}$) of one’s own voice (speaking-induced suppression, SIS [2–4]). However, the $f_{\text{0}}$ we objectively self-generate via vocal fold movements is measurably different from the $f_{\text{0}}$ we subjectively perceive as our own, due to skull bone density and air medium perceptual filtering effects [5]. We asked if the effects of repeated exposure to sensory suppression would transfer also to attentive listening to others speaking with our own objective or subjective voice $f_{\text{0}}$. To answer this question, we exploited a phenomenon known as auditory change deafness, that is the failure to detect supra-threshold auditory changes. In other words, the change is missed despite it being acoustically salient. Change deafness has been studied in multiple domains including speech, music and auditory scenes [6–10] and has been previously used to investigate $f_{\text{0}}$ properties. In a study by Gregg & Samuel [7], participants were presented with two auditory scenes of naturalistic sounds and rated them as same or different. Results showed that change deafness was strongly driven by both the fundamental frequency $f_{\text{0}}$ and the spectral content of the target stimulus, also known as harmonicity, but not by stimulus familiarity. Hence, humans rely on both the $f_{\text{0}}$ and harmonic content of a stimulus to detect changes. This conclusion was tested by McPherson & McDermott [11], building on the assumption that pitch representations, tied to their fundamental frequencies, are invariant to spectral changes [12] and should therefore be valuable for perceiving and discriminating spectrally distinct sounds. Their study used pairs of spoken vowels, instrument notes and complex synthetic sounds in a low/high paradigm. Additionally, the sounds were modified to manipulate their harmonic content or to create inharmonic versions. While results show limited pitch invariance, the authors also found that the full spectral content largely biases pitch judgements even when extreme spectral differences were introduced to force reliance on $f_{\text{0}}$ information, indicating that $f_{\text{0}}$ representations are unbiased but secondary in importance in making pitch comparisons (see also [13,14]). The dynamics of the highlighted perceptual bias also depended on the pitch difference between the first and second tones, as well as a congruence effect between their harmonics and the direction of $f_{\text{0}}$ change. $f_{\text{0}}$-based pitch judgements being the main focus of our experiment, we restricted our investigation to $f_{\text{0}}$ effects only, by employing pure tone stimuli.

Deriving distinguishable estimates for the objectively uttered and subjectively perceived $f_{\text{0}}$ pitches poses relevant experimental challenges. The involvement of both bone and air conduction (BC and AC) when hearing oneself speak requires dealing with the effects of different sound filters: besides air and bone, also skin and soft tissues. BC was shown to differ from AC in its loudness function [15] and the attenuation characteristics of BC depend on both the frequency and the location of stimulation [16,17]. Reinfeldt *et al*. [18] attempted to quantify the ratio between the AC and BC components of one’s own voice using 10 phonemes, varied between vowels and consonants. Their results show relative dominance of BC in the frequency range 1–2 kHz, with strong dependency on the vocalized phoneme and comparable results for phonemes with similar vocalization. Stenfelt & Håkansson [15] used a bone transducer to compare the loudness functions of AC and BC sound conduction pathways at different frequencies in the range 0.25–4 kHz. Their findings showed a frequency-dependent difference that reached 6–10 dB for frequencies between 250 and 750 Hz, the lower end of which overlaps with the upper limits of the frequency range of human voice $f_{\text{0}}$ in our sample. Since the physical properties of bone-conducted sound lend the BC component higher salience, it would seem appropriate to infer that subjective pitches should be generally perceived as being lower in frequency than objective ones. Yet, the results of various attempts at recreating one’s own voice challenge such conclusion. While Shuster & Durrant [19] confirmed a preference to low-pass filtered voice in a delayed-feedback paradigm that looked into 10 frequency bands in the range 0.1–6.3 kHz, Kimura & Yotsumoto [5] found high inter-subject variability when participants compared and rated five recorded samples—the unadjusted recorded own voice and four filtered samples of it—based on their similarity to their self-perception. The authors conclude that no generic transfer function can be determined to approximate with sufficient fidelity the subjective perception of voice pitch. However, they found a high rank correlation of intra-subject ratings from two different sessions (ρ = 0.899), indicating that the subjective perception of one’s own voice is relatively stable over time. If both objective and subjective voice perception processes are intra-individually stable in time, then there should exist a transfer function acting as an individual and somewhat idiosyncratic prior, which would largely account for the relationship between objective and subjective voice pitch. This would support the hypothesis that change deafness effects in the frequency neighbourhoods of objective and subjective voice $f_{\text{0}}$ should not differ.

Following the concepts presented above, if SIS brings about long-term sensory attenuation effects (perhaps mediated by repetition suppression [20–22], it might significantly alter the sensitivity to auditory change in the frequency neighbourhood of individual objective voice $f_{\text{0}}$, relative to a control frequency (Hypothesis 1). Assuming that the human brain holds a spectrotemporal filter encoding the transfer function required to compute subjective voice $f_{\text{0}}$ from the objective one, then the behavioural change deafness profiles for objective and subjective voice pitches should be affected to a similar extent (Hypothesis 2).

## 2. Material and methods

### 2.1. Participants

Fifty individuals (26 female, 24 male, 0 non-binary/diverse) between the ages of 18 and 31 years (*m* = 24.6, s.d. = 2.8) were recruited to participate in a series of auditory tests. A subset of the participants (*n* = 9, 4 female, 5 male, 0 non-binary/diverse), aged between 19 and 29 years (*m* = 24, s.d. = 2.94), was selected to test the reliability of objective voice pitch estimation methods (Experiment I). The remaining 41 participants took part in the main experiment (Experiment II); of these, 27 participants (15 female, 12 male, 0 non-binary/diverse) aged 19−29 years old (*m* = 24.5, s.d. = 2.7) completed the experiment and were thus eligible for further analysis. None of the participants reported having any hearing impairments. Additionally, their hearing was tested using a conventional scanning with an audiometer. Pure tones at 0.125, 0.25, 0.5, 0.75, 1, 2 and 4 kHz were delivered mono-aurally at 20 dBHL. The tones were presented manually and continuously until detection or for a maximum of a few seconds (5 s). Left-right-ear order was randomized.

### 2.2. Experiment I: objective pitch estimation

#### 2.2.1. Design and procedure of experiment I

Participants were asked to vocalize the vowel {ä:} three times, with each iteration lasting about 10 s. The speech sound {ä:} is an open, central, unrounded vowel, common to an array of languages including Swedish and German. A study by Reinfeldt *et al.* [18] demonstrated that the measured sound pressure in the ear canal while vocalizing the phoneme /a/ has the lowest BC-to-AC ratio among the reported phonemes. This result, along with its central place in the German language, supports the use of this vowel sound to reduce perceptual variability that might arise from idiosyncratic anatomical differences, e.g. the size or shape of the skull. Additionally, recordings were made in a continuous speech condition and a read-out-loud condition. The session lasted 30 min, during which participants performed all three conditions in a counterbalanced order. For the reading condition, an excerpt of Kafka’s ‘Die Verwandlung’ was read for a duration of 5 min. For the speaking condition, eight topics were made available for participants to choose from, and talk about. Participants were given the time to prepare beforehand and choose any number of the proposed topics, as long as they would continuously speak for 5 min. The recordings were made using a directional microphone placed about 2 m away from the seated participant. Participants were instructed to face the microphone’s general direction and to maintain a relaxed posture while speaking. They were also made aware that the microphone was sufficiently sensitive, and that they should refrain from attempting to compensate for the distance by raising their voice pitch or volume. All data were recorded as WAV files with a 48 kHz sampling frequency and 24-bit sample size, and additional downsampled copies were created in Praat (at 8, 4 and 2 kHz). The original recordings and the copies were then run through two pitch detection algorithms (PDAs), HelperPitchTracker (HPT) and Praat, to compare their outputs.

HPT is a MATLAB PDA that utilizes multiple pitch estimations, median smoothing and a hidden Markov model (HMM) [23]. It is claimed to produce robust pitch estimations even in noisy conditions [23]. We compared its performance in objective voice pitch estimation with that of Praat [24], a commonly used software package for speech and audio analysis, on the same recorded data. Praat’s pitch extraction method relies on autocorrelation [25,26].

### 2.3. Experiment II: change deafness

#### 2.3.1. Design and procedure of experiment II

The experiment consisted of four parts, carried out in one session that lasted between one and a half to two hours.

- **Estimating objective pitch values.** Participants vocalized the vowel {ä:} in their neutral, average voice. Three recordings of about 7 s were obtained with a sampling rate of 8 kHz and a sample size of 24 bits. The objective pitch estimate of a given participant corresponded to the average of three estimates extracted from their recordings using HPT.
- **Just-noticeable differences (JNDs**). The average estimate of the objective pitch, centred in a [−15 +15] Hz frequency range, was used in an adaptive staircase approach to derive each participant’s JND, between the objective pitch and the next perceptually different frequency in both directions. In each trial, two sinusoidal pure tones were presented, and participants were required to select the higher tone in terms of frequency. While one of the tones always corresponded to the objective pitch estimate, the order of presentation was randomized. There were three possible responses (tone1, tone2 or difference undetectable) resulting in one of two outcomes on each trial; the answer is correct and frequency difference is decreased on the next trial or it is incorrect/undetectable and the difference is increased. Each staircase (block) started at either limit of the frequency range (−15 or +15 Hz) and ended after 12 reversals (change from decrease to increase or vice versa). The median of the last 8 out of 12 reversals was taken to approximate the JND. Two blocks were run for each limit (direction), amounting to four in total. The result of each block, i.e. the distance between the calculated median and the objective pitch, was computed in Hz and averaged along with the rest of the blocks to obtain the final JND value.
- **Estimating subjective pitch values.** In order to infer an estimate of the subjective pitch, an indirect way of measurement was deployed. In this rather demanding task, the objective pitch estimate and the JND were utilized to create an external reference pitch, adaptable by the participant to the purpose of comparison with their own subjective pitch. Upon starting, a 2 s pure sinusoidal tone in the objective pitch frequency is played. The frequency, which was shown on screen to each participant, could be manipulated in increments equal to the JND to play similar tones at higher and lower frequencies. Additionally, tones at the current frequency could be replayed if required. Participants were again instructed to vocalize the vowel {ä:} in their neutral, average voice right before or during every single-tone presentation. Their goal was to find the frequency that matched their subjective perception of their voice as closely as possible. Participants were not explicitly informed that the initial frequency was their own but were told that they should explore the frequency space for as long as needed before making their decision. Due to the apparent difficulty in keeping a constant reference pitch over multiple comparisons, extensively practising a neutral, comfortable and consistent vocalization of the vowel at the beginning of the experiment was highly encouraged.
- **Frequency change deafness task.** An oddball paradigm was designed with 180 pure tone sequences, divided into three conditions according to their base frequency (subjective, objective and control). A control frequency was chosen for each individual participant by shifting the subjective pitch frequency by +50 (male) or −50 Hz (female). Each sequence consisted of 18 sinusoidal pure tones of a randomized duration between 200 and 600 ms in steps of 50 (200:50:600), separated by 17 silences (5:5:45) ms. Each tone was passed through a Tukey window with a 0.05 ms taper width. Using a 0.5 probability of a sequence having a deviant tone, each condition contained 30 sequences with one deviating tone and 30 without any deviants. Within a given condition, deviant sequences were further broken down into six subgroups of five sequences each to cover six possible deviation magnitudes, which were defined as [−3S, −2S, -S, S, 2S, 3S], where S (for step) is derived per participant as follows: S=|subjective−objective|/3 and S≥minJND. If S<minJND, the staircase results were inspected for possible procedural issues such as faulty hardware or task ambiguity, then the parts from estimating the JND were repeated (see table 7 in appendix C for exact values). Should S<minJND have remained true, the experiment was terminated and the dataset discarded. Finally, deviant tones occurred at a randomly selected position within the latter half of its sequence. Participants were instructed to listen to each sequence and report whether a deviant tone was present or not within a sequence. The generated sequences (*n* = 180) were randomly assigned to six blocks of 30 sequences each.

#### 2.3.2. Analysis of experiment II

**Figure 1.**
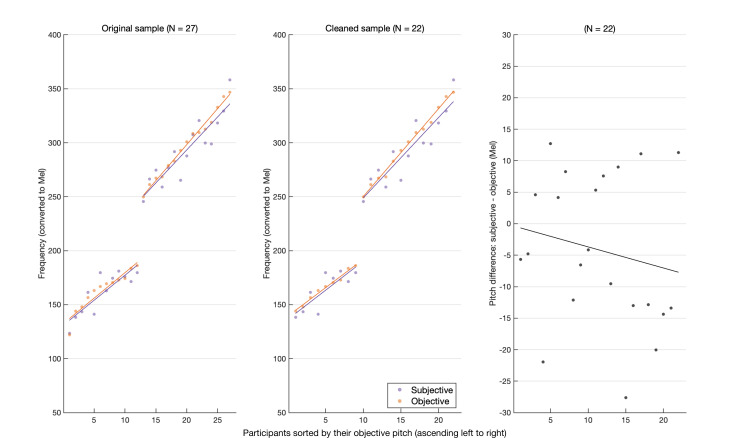
Participants sorted ascendingly by the objective pitch estimate. (*left*) The objective and subjective pitch estimates per participant (*n* = 27). (*middle*) The same plot for the filtered sample (*n* = 22). (*right*) The difference between both estimates per participant scales up, albeit only moderately, with the measured pitch estimates. The dichotomy (high and low clusters) codes for participants' reported sex. The estimates where measured in Hz and converted to the Mel scale to simplify comparisons in this figure.

Twenty-seven participants out of the initial 41 completed all four parts of the change detection experiment. *d*’ sensitivity estimates were calculated for each pitch condition. A *d*’ cut-off value was computed by simulating a binomial distribution (*n* = 10 000) with 60 trials at a 0.5 target probability. The cut-off was at $d^{\prime }\approx$ 0.51 and five participants were excluded for falling below it in one or more pitch conditions. The final sample (*n* = 22, 9 male, 13 female, 0 non-binary/diverse) was between 19 and 29 years old (*m* = 24.45, s.d. = 2.63). The pitch estimates ranged between 95.4 and 252.3 Hz for the objective pitch (*m* = 171.25, s.d. = 55.29) and 91.4 and 261.9 Hz for the subjective pitch (*m* = 167.97, s.d. = 54.26). The absolute average pitch difference was 8.52 Hz (s.d. = 4.89), translating to 0.9 semitones (s.d. = 0.54). Figure 1 illustrates the similarity between the subjective and objective pitch and the difference between them, transformed into the Mel scale to conveniently depict the magnitude of pitch differences in perceptual terms.

**Figure 2.**
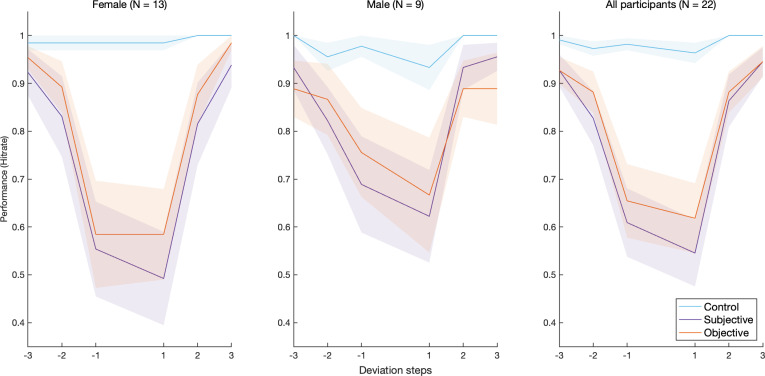
Change detection hit rate for each deviation step per pitch condition, averaged across participants. Shaded area is the s.e.m. (*left*, *middle*) Average hit rate broken down by participants’ reported sex. (*right*) Average hit rate for the whole sample.

**Figure 3.**
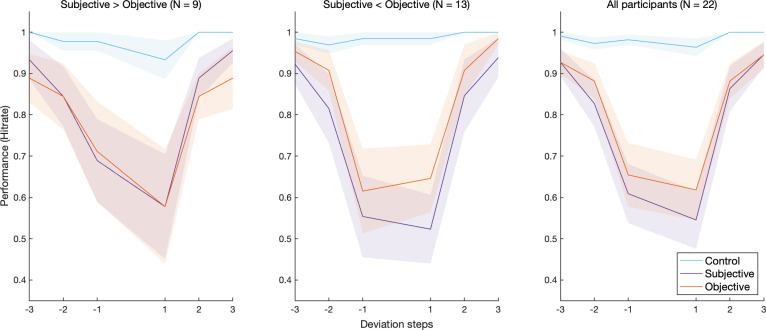
Change detection hit rate for each deviation step per pitch condition, averaged across participants. Shaded area is the s.e.m. (*left*, *middle*) Average hit rate broken down by the relative position of the subjective pitch estimate to the objective pitch estimate; Subjective is higher vs Objective is higher. (*right*) Average hit rate for the whole sample.

A signal detection theory (SDT) analysis was implemented to compute the hit rate in each deviation step per condition. Additionally, a general mixed-effects linear model (GLMM) was constructed to contrast performance between conditions. Hit rates were calculated for each deviation step in each condition and averaged over all of the participants. Figure 2 and 3 [27] show the average hit rate for each deviation step—excluding zero—colour-coded by pitch condition. Figure 2 shows the performance of male vs female participants, reflecting their underlying frequency clusters. Figure 3 splits the sample into two groups based on the sign of the difference (Subjective—Objective), i.e. the relative pitch position (compare figure 1, middle and right panels). Note: a more conservative cut-off (*d*’ = 1) introduced no changes to the final sample and the subsequent analysis.

#### 2.3.3. Model factors

Figure 2 and 3 seem to be in concordance with Hypothesis 1 and, to a lesser extent, Hypothesis 2 (see §1). We attempted to quantify the effects of pitch condition and deviation magnitude within each condition on performance. Moreover, the figures show distinct behavioural patterns in the subsamples of reported biological sex and relative pitch position, which were therefore included in the model. Additionally, inter-subject variability in response to different pitch conditions and deviation magnitudes was taken into account. Thus, we constructed the following maximal random-slope random-intercept model that accommodated the data as well as possible, without overstepping the theoretical bounds of our stated hypotheses:

$m_{max}$: Response = Condition * Magnitude * Relative Position * Sex + (1 + Condition * Magnitude | Subject)

Overall, the model consisted of four fixed effects and one random-effects term, the fixed effects being the following: Condition, referring to the three experimental $f_{\text{0}}$ conditions, Subjective, Objective, and Control; Magnitude with six levels comprising the negative and positive deviation steps (excluding zero); Relative Position with two levels, SubjectiveAbove or SubjectiveBelow to code the spectral positioning of the subjective pitch $f_{\text{0}}$ relative to the objective pitch $f_{\text{0}}$; and finally, reported biological Sex with two levels for male and female participants. The random-effects term comprised a random intercept for Subject, with 22 levels coded by the participant’s number, and random slopes for Condition and Magnitude. By design, Subject was nested in both Sex and Relative Position and these two variables were crossed. Combinations of the different factor levels of the four fixed-effect factors amounted to 72 unique cells, the responses in 21 of which had zero variance due to ceiling performance. Zero variance cells lead to anomalies in computing confidence intervals of the estimated marginal means (see §3.2.2) due to the way these intervals are calculated on the logit scale and back-transformed to the response scale. The issue was tackled, prior to model simplification and selection, by randomly introducing artificial errors to the mentioned cells, i.e. by replacing one randomly selected hit with a miss. Notably, the cells were almost equally split between the grouping factors Sex and Relative Position ($N_{Sex}$ = 9 and 12, female and male; $N_{RelPos}$= 10 and 11, SubjectiveAbove and SubjectiveBelow). Additionally, most of the cells with zero-variance and ceiling performance occurred in the control condition ($N_{Control}$ = 16). The model was fitted with the R package (lme4) [28] to the binomial, trial-by-trial response data obtained from the frequency change deafness task.

#### 2.3.4. Model simplification and selection

The simplification process was carried out in a step-wise fashion, starting with the random-slope term. As soon as a converging model was reached, the final random term, a random-intercept for Subject (1 | Subject) was adopted. The simplification of the fixed terms is followed by progressively dropping insignificant effects from the model to reach a minimum Akaike information criterion (AIC). Wald chi-square (ANOVA type II) tests were run on every intermediate model and insignificant effects were dropped in a descending order, from high-order interactions towards main effects. Once a minimum AIC was reached—at which further simplification moved AIC away from its minimum—the initial complex random-effect term was re-plugged and simplified again.

The selection process between the final candidates (table 1) judged the models based on their AIC and ability to replicate the data, the latter evaluated in terms of the model’s conditional Nakagawa’s $R^{2}$. Between-model ANOVA tests showed that $m5_{RS2}$ was significantly better than the competing models at a 5% significance level, including $m5_{RI}$ with the same fixed-effect structure and a random-intercept for Subject. The model $m5_{RS2}$ had a lower AIC than the other candidates, and a higher conditional coefficient of determination $R_{cond}^{2}$= 0.567 [$CI_{95\%}$: 0.501, 0.768].

**Table 1.** An overview of the potential models and their selection criteria during model simplification and selection. Resp = response, Cond = pitch condition, Mag = deviation magnitude, RelPos = relative pitch position; RI = random intercept, RS = random slope, in reference to the random-effect term.

model	AIC	$R_{cond}^{2}$
$m0_{RI}$ : Resp = Cond * Mag * RelPos * Sex + (1 | Subject)	1274.6	0.559
$m1_{RI}$ : Resp = Cond * Mag * RelPos + (1 | Subject)	1237.4	0.541
$m2_{RI}$ : Resp = Cond * Mag * Sex + (1 | Subject)	1230.4	0.555
$m5_{RI}$ : Resp = Cond * Mag + (1 | Subject)	1218.6	0.526
$m5_{RS2}$ : Resp = Cond * Mag + (1 + Cond | Subject)	1195.9	0.567

#### 2.3.5. Model diagnostics

Several diagnostic tests were run on the model using simulated scaled residuals (*n* = 10 000 simulations) created by the ‘DHARMa’ package as illustrated by Hartig & Lohse [29]. The residuals were created by simulating new data from the current fit, including the random effects, and comparing the simulated data with the observed data (no refitting). Prior to the simulation, overdispersion was assessed with the ‘performance’ package [30] (ratio = 0.730, ${\tilde{\chi }}^{2}$= 1427.16, *p* = 0.1). The absence of overdispersion was further confirmed by a dispersion test using ‘DHARMa’ (ratio = 0.994, *p* = 0.98). Additionally, an asymptotic one-sample Kolmogorov–Smirnov (KS) test of uniformity was run (*D* = 0.011, *p* = 0.96).

## 3. Results

### 3.1. Results of experiment I

HPT ran out of memory while processing the speaking and reading recordings at 48 kHz. The algorithm also faced difficulties calculating pitch estimates for some of the 2 and 4 kHz recordings. This resulted in a number of missing data points and two abnormally inflated values. HPT successfully extracted pitch estimates for all files for the 8 kHz sampling frequency recordings. Praat successfully extracted pitch estimates for all samples and sampling frequencies. However, upon closer examination, a number of the vowel samples processed in Praat showed U-shaped drops in their resulting pitch contours, which considerably lowered their respective pitch estimates. The drops were edited out manually and corrected estimates were calculated to be used in the analysis (see appendix A for details of these findings and the correction approach). From this point onwards, we report on the analysis of the 8 kHz sampling rate datasets. Voice pitch estimates were pooled into one data array per extraction algorithm. Each array included the pitch estimates acquired from all participants across the three conditions, amounting to 27 data points per extraction algorithm: nine for reading, nine for speaking and nine for the vowel task, the last being the mean estimate of all three vowel iterations per participant. Both data arrays were normally distributed ($D_{\alpha =0.05}$ = 0.17, *p* = 0.4 and $D_{\alpha =0.05}$ = 0.18, *p* = 0.03, for estimates of Praat and HPT, respectively).

**Figure 4.**
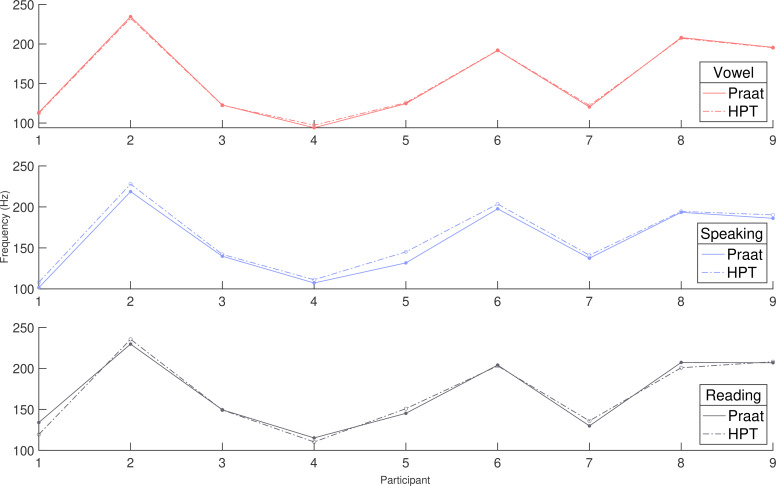
Fundamental pitch estimates in Hz for each condition (reading, speaking and vowel) from both PDAs per participant.

**Figure 5.**
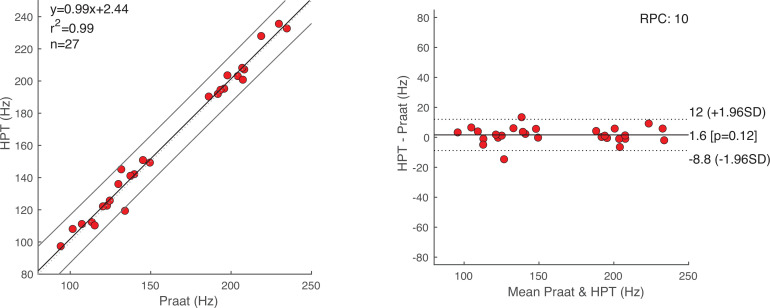
(*left*) Correlation plot of Praat's and HPT's $f_{\text{0}}$ estimates of all three voicing conditions (reading, speaking and corrected vowel) across participants. (*right*) Bland–Altman LOA plot with 95% CIs. The difference between HPT and Praat for a given participant in a given condition is plotted on the y-axis against the average of both estimates on the x-axis.

**Figure 6.**
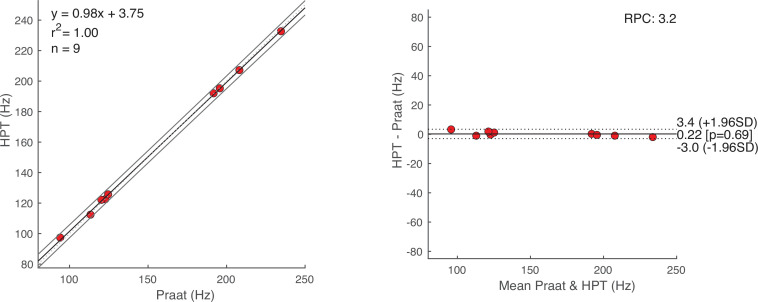
(*left*) Correlation plot of Praat's & HPT's $f_{\text{0}}$ estimates of the corrected vowel a: condition. (*right*) Bland–Altman LOA plot with 95% CIs. The ordinate and abscissa represent the same as in figure 5.

The product-moment correlation coefficient between PDAs for each condition (task) was very high (all *r* ≥ 0.99, *p* ≪ $10^{-3}$), indicating a strong correlation between HPT and Praat at 8 Hz sampling rate (figure 4). Since the relatively large data range can lead to numerical inconsistencies when calculating the correlation coefficients, we opted for measuring the agreement of methods as outlined by Bland & Altman [31]. Every data point in figure 5 (right panel) [32] represents the result of the subtraction of a pair of estimates from the two algorithms (HPT—Praat), plotted against the mean of that pair. The differences (HPT—Praat)—across conditions—(m = 1.64, s.d. = 5.31 Hz) come from a normal distribution ($D_{\alpha =0.05}$ = 0.16, *p* = 0.45) with a mean of zero ($t_{\left(df=26,\alpha =0.05\right)}$ = 1.6, *p* = 0.12). The same assumptions were tested for PDA subtractions within each condition; reading (m = −0.92, s.d. = 6.88 Hz; $D_{\alpha =0.05}$ = 0.16, *p* = 0.94; $t_{\left(df=8,\alpha =0.05\right)}$ = −0.4, *p* = 0.7), speaking (m = 5.6, s.d. = 3.79 Hz; $D_{\alpha =0.05}$= 0.2, *p* = 0.82; $t_{\left(df=8,\alpha =0.05\right)}$ = 4.43, *p* = 0.002) and vowels (m = 0.22, s.d. = 1.63 Hz; $D_{\alpha =0.05}$ = 0.17, *p* = 0.92; $t_{\left(df=8,\alpha =0.05\right)}$ = 0.41, *p* = 0.69). Figure 5 (right panel) also shows the 95% limits of agreement (LOA) in the range ± 1.96 s.d. [−8.8, 12] Hz difference between the PDAs across conditions. The reproducibility coefficient (RPC) suggests that the difference between a random pair of future estimates, measured by the two evaluated PDAs, will fall within 10 Hz with 95% probability. Applying the LOA method to the three conditions separately shows a much higher level of agreement for the vowel condition (figure 6), compared with reading and speaking (in Hz: m= 0.22, s.d. = 1.63; range ± 1.96 s.d. [−3, +3.4]; RPC = 3.2; and $D_{\alpha =0.05}$ = 0.17, *p* > 0.92). We conclude that individual voice pitch estimates can be reliably obtained in the simplest of conditions by vocalizing the vowel {ä:}. We also judge the estimates of HPT to be reliable and its performance comparable to that of Praat in the setting of this investigation, making it suitable to automate the pitch estimation and stimuli creation process without the need for human intervention. For LOA plots of the other two conditions, as well as the uncorrected vowel estimates, see Appendix B (figures 11–13) and appendix A (figure 10), respectively.

### 3.2. Results of experiment II

#### 3.2.1. Model results

**Figure 7.**
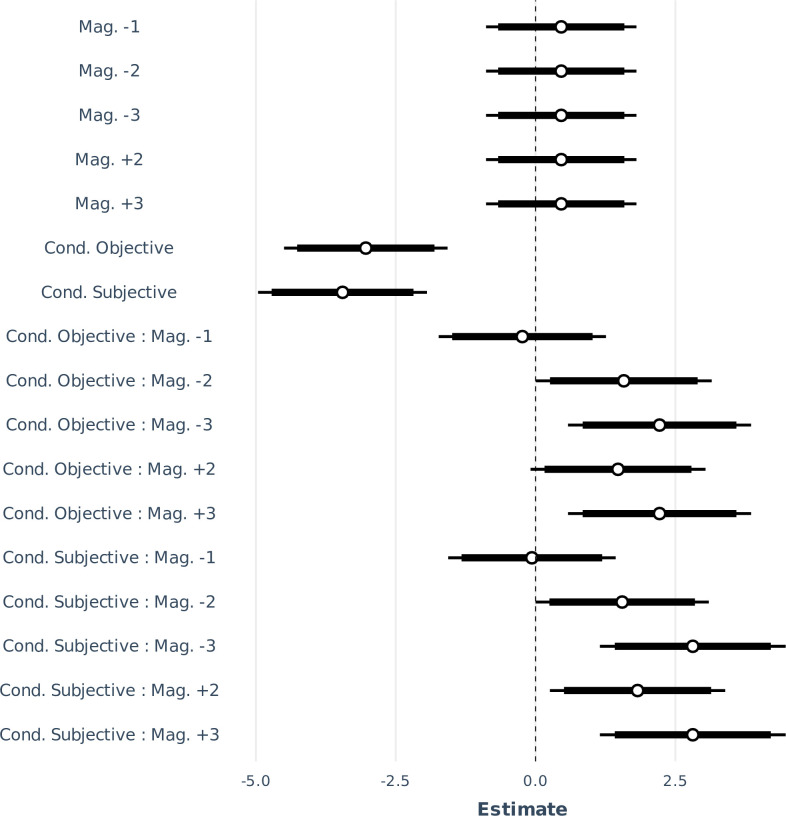
Fixed-effects estimates and their interactions of the model $m5_{RS2}$ with 95 and 90% CIs, indicated in thin and thick lines, respectively.

**Figure 8.**
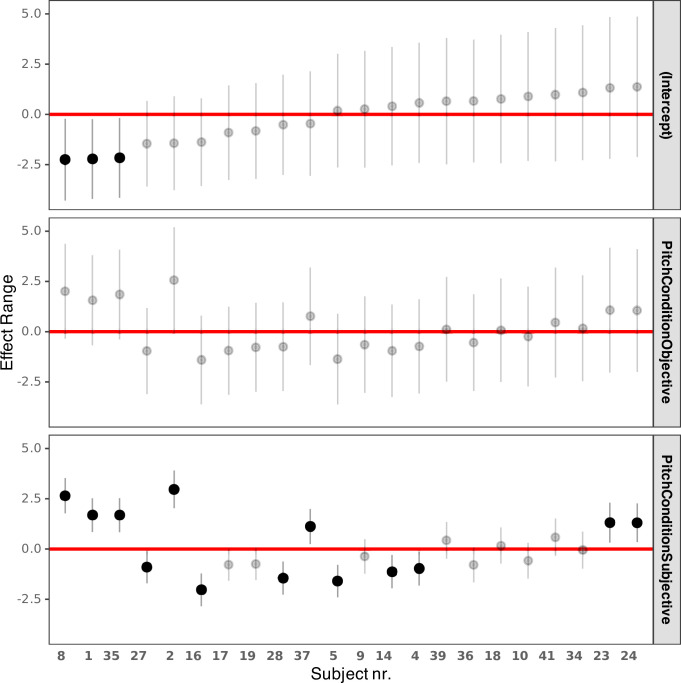
Random-intercept and random-slope variance per pitch condition within each subject, referenced to the control pitch condition.

Tables 2 and 3 show the coefficients of the fixed and random effects of the final model $m5_{RS2}$. *p*-values were omitted from the fixed effects table as they are considered misleading. Instead, their unexponentiated estimates were visualized, using the ‘jtools’ package [33], with 90% and 95% CIs in figure 7. The residual variance of the random effect is not reported as it does not apply in GLMMs. Figure 8 illustrates the response variance explained by the random effects, grouped by subject. The responses varied more strongly in the subjective pitch condition, within the same subject. It is difficult to confirm whether the increased variance in the subjective pitch condition is due to perceptual factors or a suboptimal estimation of the subjective pitch fundamental frequency, which was used to construct the stimuli in this condition.

**Table 2.** Exponentiated estimates of the fixed effects coefficients of $m5_{RS2}$ . Cond. = pitch condition, Mag. = deviation magnitude.

	exp(Est.)	s.e.	z val.
(intercept)	42.37	0.71	5.31
Cond. Objective	0.05	0.75	−4.07
Cond. Subjective	0.03	0.77	−4.48
Mag. −1	1.58	0.69	0.67
Mag.+2	1.58	0.69	0.67
Mag. −2	1.58	0.69	0.67
Mag.+3	1.58	0.69	0.67
Mag. −3	1.58	0.69	0.67
Cond. Objective:Mag. −1	0.79	0.76	−0.31
Cond. Subjective:Mag. −1	0.94	0.76	−0.09
Cond. Objective:Mag.+2	4.36	0.80	1.85
Cond. Subjective:Mag.+2	6.19	0.80	2.28
Cond. Objective:Mag. −2	4.84	0.80	1.97
Cond. Subjective:Mag. −2	4.70	0.79	1.96
Cond. Objective:Mag.+3	9.17	0.83	2.65
Cond. Subjective:Mag.+3	16.61	0.85	3.32
Cond. Objective:Mag. −3	9.17	0.83	2.65
Cond. Subjective:Mag. −3	16.61	0.85	3.32

**Table 3.** Random-slope variation per subject, with a random-intercept per pitch condition, referenced to the control pitch condition. Participants show a higher variance in responses in the subjective pitch condition.

group	parameter	s.d.
subject	(intercept)	1.56
	Cond. Objective	1.55
	Cond. Subjective	1.79

#### 3.2.2. Marginal means and contrasts

**Figure 9.**
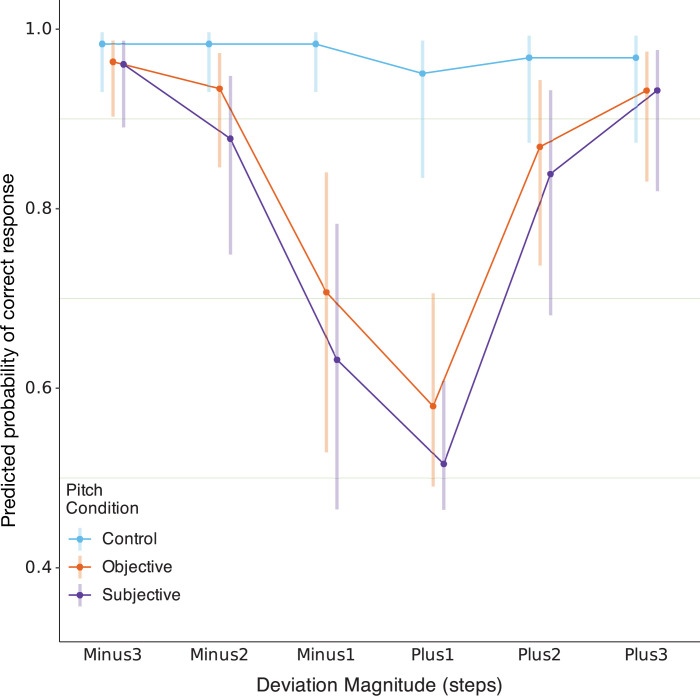
The estimated marginal means as computed from the final model $m5_{RS2}$. The plot shows the estimates for each pitch condition in every deviation magnitude. The bars indicate asymptotic 95% CIs. Both the estimates and the intervals are shown on the response scale (hit rate), back-transformed from the logit scale with bias-adjustment.

**Figure 10.**
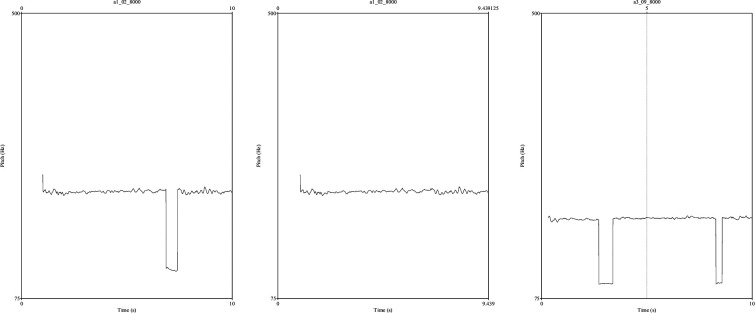
(*left*) Example of the drops in Praat-generated pitch contours of the first vowel recording from participant no. 2 (before manual correction). (*middle*) The same vowel after manual contour correction. (*right*) Example of multiple contour drops form in the third vowel recording from participant no. 9.

**Figure 11.**
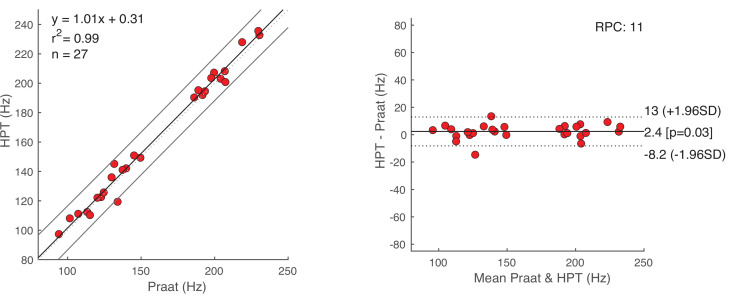
(*left*) Correlation plot of Praat's & HPT's $f_{\text{0}}$ estimates of all three voicing conditions (reading, speaking and vowel) across participants. (*right*) Bland–Altman LOA plot with 95% CIs. The ordinate and abscissa represent the same as in figure 5.

**Figure 12.**
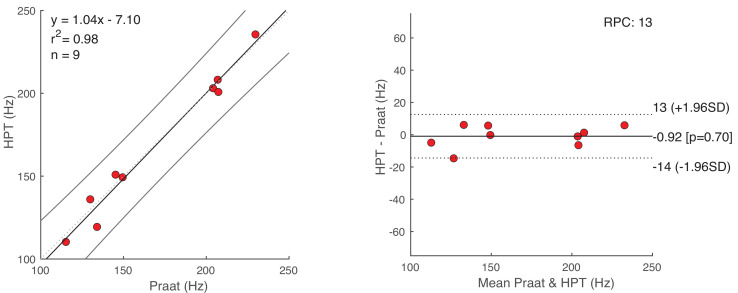
(*left*) Correlation plot of Praat's and HPT's $f_{\text{0}}$ estimates of the reading condition. (*right*) Bland–Altman LOA plot with 95% CIs. The ordinate and abscissa represent the same as in figure 5.

**Figure 13.**
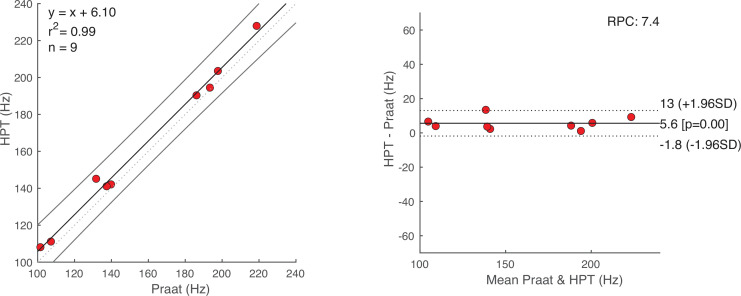
(*left*) Correlation plot of Praat's and HPT's $f_{\text{0}}$ estimates of the speaking condition. (*right*) Bland–Altman LOA plot with 95% CIs. The ordinate and abscissa represent the same as in figure 5.

The model $m5_{RS2}$ showed strong main effects of the deviation magnitude and pitch condition (table 4) and a significant condition–magnitude interaction. The estimated marginal means of the predicted hit rate in each deviation magnitude within each pitch condition were calculated (table 5) and plotted (figure 9). The model adequately reflected the behavioural patterns observed in the data (figures 2 and 3), capturing the sharp drop in performance for the subjective and objective conditions, in contrast with a near-ceiling performance in the control condition. In line with our predictions, the effects are especially pronounced for the ±1 deviation magnitudes, i.e. the immediate vicinity of the fundamental frequency of the own voice. Pairwise contrasts between pitch conditions at each deviation magnitude further confirmed the significance of these effects at ±1 for the subjective and objective conditions (table 6). No significant subjective–objective contrasts could be found in any of the deviation magnitudes.

**Table 4.** The results of the Wald chi-square test on $m5_{RS2}$. It shows a strong main effect for deviation magnitude, about an order of magnitude larger than that of the pitch condition, as well as a significant interaction between both factors.

	chi-sq.	d.f.	Pr( > chi-sq.)
pitch condition	12.3	2	0.002
deviation magnitude	129.73	5	<2.2e−16
pitch condition:deviation magnitude	23.66	10	0.009

**Table 5.** The estimated marginal means for each pitch condition by deviation magnitude (± step), as computed from the model $m5_{RS2}$ . The estimated marginal means are expressed in the ‘prob’ column and are given a scale [0,1] after back-transforming them from the logit scale. ‘prob’ refers to the probability of obtaining a correct response (a hit). LCL and UCL are asymptotic confidence intervals at 95%. Estimates and confidence intervals are adjusted for bias.

magnitude	condition	prob	s.e.	LCL	UCL
Plus1	control	0.98	0.02	0.89	1.00
	objective	0.67	0.09	0.43	0.84
	subjective	0.57	0.11	0.32	0.79
Minus1	control	0.99	0.01	0.91	1.00
	objective	0.72	0.08	0.49	0.87
	subjective	0.67	0.10	0.41	0.85
Plus2	control	0.99	0.01	0.91	1.00
	objective	0.93	0.03	0.82	0.98
	subjective	0.93	0.03	0.80	0.98
Minus2	control	0.99	0.01	0.91	1.00
	objective	0.94	0.03	0.83	0.98
	subjective	0.91	0.04	0.76	0.97
Plus3	control	0.99	0.01	0.91	1.00
	objective	0.97	0.02	0.89	0.99
	subjective	0.97	0.02	0.90	0.99
Minus3	control	0.99	0.01	0.91	1.00
	objective	0.97	0.02	0.89	0.99
	subjective	0.97	0.02	0.90	0.99

**Table 6.** Table of contrasts. Small pitch deviations of ±1 step from $f_{\text{0}}$ were significantly more likely to be detected in the control pitch condition, compared with the subjective and objective pitch conditions. The contrast estimates are given on the log odds ratio scale. LCL and UCL are asymptotic confidence intervals at 95%. *p*-values and confidence intervals are Bonferroni-adjusted for hree tests and three estimates, respectively. Pitch condition abbreviations: ctrl. = control, obj. = objective, and subj. = subjective.

magnitude	contrast	estimate	s.e.	LCL	UCL	z ratio	*p*
Plus1	Ctrl.—Obj.	3.04	0.75	1.25	4.82	4.07	0.0001
	Ctrl.—Subj.	3.45	0.77	1.60	5.29	4.48	<0.0001
	Obj.—Subj.	0.42	0.36	−0.44	1.27	1.16	0.74
Minus1	Ctrl.—Obj.	3.27	0.81	1.34	5.21	4.05	0.0002
	Ctrl.—Subj.	3.52	0.83	1.53	5.51	4.23	0.0001
	Obj.—Subj.	0.24	0.37	−0.64	1.12	0.67	1
Plus2	Ctrl.—Obj.	1.56	0.85	−0.46	3.58	1.85	0.19
	Ctrl.—Subj.	1.63	0.86	−0.44	3.70	1.88	0.18
	Obj.—Subj.	0.07	0.50	−1.14	1.27	0.13	1
Minus2	Ctrl.—Obj.	1.46	0.85	−0.57	3.49	1.72	0.26
	Ctrl.—Subj.	1.90	0.86	−0.15	3.95	2.22	0.08
	Obj.—Subj.	0.44	0.50	−0.75	1.63	0.90	1
Plus3	Ctrl.—Obj.	0.82	0.88	−1.29	2.93	0.93	1
	Ctrl.—Subj.	0.64	0.91	−1.54	2.82	0.71	1
	Obj.—Subj.	−0.18	0.63	−1.69	1.33	−0.28	1
Minus3	Ctrl.—Obj.	0.82	0.88	−1.29	2.93	0.93	1
	Ctrl.—Subj.	0.64	0.91	−1.54	2.82	0.71	1
	Obj.—Subj.	−0.18	0.63	−1.69	1.33	−0.28	1

## 4. Discussion

We used a change deafness paradigm to test the sensitivity to frequency change during listening for both objectively self-produced and subjectively perceived fundamental frequencies of one’s own voice. We found significant change deafness effects in the frequency neighbourhood of both objective and subjective voice pitches, compared with a control frequency. There was no significant difference between subjective and objective voice pitch change deafness profiles. We interpret these findings within the action-specific suppression framework of the CD signal [3,34,35], and suggest that reduced frequency resolution in the vicinity of the frequencies of our own voice during attentive listening may be a long-term consequence of auditory neural response suppression while speaking. SIS effects at the level of the auditory cortex may occur simultaneously for both objective and subjective voice pitches, as the brain can rely on prior knowledge of the bodily filter effects, and thus operate an additional computation on the CD. If so, then the phenomenological space of the sense of agency while speaking should be rethought as including one’s own body and the immediate environment as relevant computational factors in determining the neural bases for the sense of self.

The frequency range and shape of change deafness effects could stem from two separate factors. First, within a CD-mediated sensory modulation perspective, speech-induced suppression (SIS) should follow the natural fluctuations in frequency around the $f_{\text{0}}$ of the own voice [36]. As a consequence, the hypothesized long-term effect of CD signals on change deafness should involve a narrow-band frequency region rather than just the $f_{\text{0}}$. If individual transfer functions are stable in time, then the suppression effects for subjective voice pitch should mimic in width those for objective voice pitch. Second, decreased frequency resolution in the surroundings of the $f_{\text{0}}$ may be a consequence of neural plasticity [37–39], subsequent to frequency-selective lateral inhibition effects [40,41]. Wu *et al.* [41] found that frequency selectivity increased due to inhibitory inputs that resulted in sharpening the tuning curve of excitatory neurons, in response to a stimulus of their characteristic frequency (CF). They also found that the inhibition stemmed from inhibitory neurons that possess a similar, yet broader, tuning curve compared with their excitatory counterparts. The inhibitory effect was also shown to increase outwards from the peak of the excitatory tuning curve, resulting in a strong suppression in the flanks of the CF. Additionally, the authors demonstrated a similar tonotopic distribution for both types of neurons. We postulate that the change deafness effect we document could be linked to long-term modulation via lateral inhibition of the CD suppression effects. Eliades & Wang [3] identified a subpopulation of auditory cortical neurons that exhibited a heightened sensitivity during vocal production, when simulating a spectrally shifted auditory feedback (also see [42]). These neurons were characterized by a suppressed baseline activity during vocalization with an unadjusted feedback. This is also compatible with Sitek *et al*.’s [43] report on variation in cortical suppression during the generation of an utterance, depending on how much it deviates from the previous utterance, i.e. its vocal context. Such behaviour constitutes a deviation from the overall suppression effects of the auditory cortex described in the literature and is linked to the role of these neurons in self-monitoring and feedback loops during vocalization. Assuming our results are robust, the work of Eliades & Wang offers a strong incentive to hypothesize that neurons with a CF that corresponds to one’s own voice fundamental frequency (and its natural variations during speech) may also belong to the population with an increased activity during self-initiated vocalization. Functionally, the high selectivity in these neurons could be deployed on demand during vocalization as a mechanism of neuronal recruitment in precise feedback loops.

Our work does not quantify the representational distance between subjective and objective $f_{\text{0}}$, and the level of potential overlap between them. Our design is not aimed at testing hypotheses about co-activation of one representation by a stimulus designed to activate the other, e.g. a tone at the frequency of subjective $f_{\text{0}}$ activating objective pitch representations (see §2.3.1). For the sake of our experiment, we assumed there exists a stable and measurable difference between objective and subjective individual voice pitch estimates. Deriving estimates of subjective voice pitch based solely on phenomenological reports is problematic, as it is difficult to verify and heavily influenced by confounds over short periods of time, e.g. the overall health of the peripheral auditory structures and the speech organs. Nevertheless, as mentioned, the transfer function between objective and subjective pitches was found to be stable intra-individually [5,44]. While these studies used filters that affected relatively wide frequency bands, Morii & Sato [45] attempted to reproduce one’s own voice by locally manipulating $f_{\text{0}}$, while preserving the harmonic content without modification. Their results show a significant increase in similarity ratings to one’s own voice by solely relying on $f_{\text{0}}$ manipulation. Our approach to derive an estimate of the subjective pitch assumes intra-individual stability in the perception of our own voice, as well as the reproducibility of a subjective estimate using $f_{\text{0}}$ manipulation. However, as a limit to our study, it is worth noting that while flattening speech stimuli and reducing them to their $f_{\text{0}}$ may be justified by a forcible reliance on $f_{\text{0}}$ information, withholding harmonic information and using only pure tones could adversely affect performance.

A further issue with our methodology concerns the absence of JND estimates at the control frequency, due to procedural limitations, particularly the experiment’s demanding nature. However, from our findings of a reduced frequency resolution near one’s own $f_{\text{0}}$, we expect the JND at the control $f_{\text{0}}$ to be lower in comparison. This assumption is supported by the near-ceiling performance in the control condition with deviations based on the JND of the objective $f_{\text{0}}$.

In summary, considering the non-uniform nature of self-generated speech suppression effects, a comprehensive model of the sense of self while speaking and listening requires taking into account both objective and subjective fundamental frequencies of our own voice. The effects we observed suggest a decrease in frequency discrimination resolution near the fundamental frequency of one’s own voice, possibly as a sustained by-product of the well-established SIS. Whether such reduced resolution has any measurable behavioural benefits impinges on (i) its transferability to spectrally more complex stimuli such as human speech, and (ii) the interaction with linguistic category and speaker identity cues, over and beyond the acoustic properties of the stimuli. For instance, Niziolek & Guenther [46] demonstrated that behavioural and cortical responses to auditory feedback alterations were significantly stronger if they crossed the phonemic category boundaries of the altered vowels, compared with sub-phonemic alterations occurring within category boundaries. Finally, it is important to situate our findings within a functional context, if only speculatively. While our study relies on well-documented mechanisms, such SIS and CD, the possibility exists that the observed effects are due to yet unexplored lifelong functional changes in the selectivity of cortical auditory responses to external stimuli, and that such changes shape also the construction of social relationships, as we might prefer to tune in to others with a voice rather different from ours.

## Data Availability

Data, metadata and the analysis script for experiment 1, as well as the model input data and the analysis script of experiment 2 are accessible on the OSF repository at the following link [47].

## Appendix A. Pitch contour correction

Praat’s PDA produced estimates for every file in every sampling frequency. However, upon inspecting the pitch contour generated for the 8 kHz vowel samples, disproportional rectangular U-shaped drops of varying duration were noticed. In total, 12 drops were found in nine vowel recordings belonging to three participants (2, 8 and 9). Eleven out of twelve drops had a duration between 150 and 690 ms (*m* = 345, s.d. = 186; 3.45 and 1.86% of a single vowel duration). The 12th drop was exceptionally longer at 1770 ms (17.7%). The average pitch estimate at the bottom contour of the rectangle was expressed as a percentage of the corrected pitch to give the drop’s magnitude. The corrected pitch is the average pitch estimate of the remaining contour after removing the drops in a given recording. These magnitudes were between 49.8 and 52.48% of the corrected pitch (*m* = 50.39, s.d. = 0.72). Performing this correction resulted in a number of changes, most notably tightening the limits of agreement and turning insignificant a previously significant *t*‐test between HPT and Praat estimates (compare figure 5 and appendix B for more details). See (figure 10) for examples.

## Appendix B. Bland–Altman LOA plots

See figures 11–13 for LOA plots of all voicing conditions (before vowel correction), as well as seaparate plots for the reading and speaking conditions.

## Appendix C. Step size and average just-noticeable difference (JND)

Table 7 lists the average JND and deviation step size in Hz for each participant in our final sample (N = 22).

**Table 7.** Averaged JND and deviation step sizes per participant in Hz. The reported JNDs are averages of four staircases that ascend/descend from ±15 Hz to the objective fundamental frequency. The condition S≥minJND was not applied to these values, but to the minimum of the four staircase JNDs before averaging, as an internal sample selection condition for participants with sufficient resolution between the fundamental frequencies of their subjective and objective pitches.

participant ID	step size (Hz)	average JND (Hz)
1	2.00	1.50
2	7.33	2.13
4	3.50	2.81
5	3.50	3.31
8	1.00	0.50
9	3.97	1.75
10	2.50	2.50
14	3.73	5.69
16	1.07	1.50
17	2.40	3.50
18	1.40	2.13
19	3.07	2.31
23	3.20	0.63
24	3.03	1.25
27	1.10	1.75
28	5.47	5.13
34	2.00	1.13
35	1.13	1.63
36	2.93	1.00
37	5.20	1.13
39	1.60	2.25
41	1.33	0.75

## Appendix D. Apparatus

See table 8 for an overview of the experiment’s setup.

**Table 8.** Technical specifications of the hardware components and software used in this study.

hardware	
microphone	Sennheiser MKE 600
physical audio interface	RME Babyface Pro FS
headphones	Beyerdynamic DT−770 Pro
	Etymotic ER3C insert earphones
recording computer	MacBook Pro 13’, early 2011
	macOS High Sierra v.10.13.6
analysis computer	MacBookPro17,1
	macOS Ventura v.13.0
	and
	MacBookPro 13’ M1, 2020
	macOS Sonoma v.14.3
software	
desktop audio interface	RME Totalmix
MATLAB (recording)	R2020a
MATLAB (analysis)	R2022a, R2023a
RStudio	2023.06.1+524
